# Tailoring lifestyle interventions to low socio-economic populations: a qualitative study

**DOI:** 10.1186/s12889-018-5877-8

**Published:** 2018-08-03

**Authors:** Nia Coupe, Sarah Cotterill, Sarah Peters

**Affiliations:** 1 Manchester Centre for Health Psychology, School of Health Sciences, The University of Manchester, Manchester, UK; 2 Centre for Biostatistics, School of Health Sciences, The University of Manchester, Manchester, UK

**Keywords:** Low socio-economic, Lifestyle interventions, Tailoring, Weight loss, Obesity

## Abstract

**Background:**

People living in deprived areas are more likely to be overweight or obese, have poorer health outcomes, and tend to benefit less from interventions than those from more affluent backgrounds. One approach to address such health inequalities is to tailor existing interventions to low socio-economic populations, yet there is limited evidence to inform their design. This study aims to identify how best to tailor lifestyle interventions to low socio-economic populations to improve outcomes.

**Methods:**

Following direct observations of community-run weight loss groups, we interviewed 11 group facilitators and 14 service users from a health improvement service in a low socio-economic area in the North West of England. Audio-recorded interviews were transcribed verbatim and analysed thematically.

**Results:**

We identified two overarching themes within the data. The first theme, managing diversity, included challenges faced in delivering a generic intervention to a diverse population in terms of knowledge, language and literacy skills, and cultural diversity. The second theme incorporated all issues relating to the environment, such as cost, access and availability of food and leisure facilities, and ‘life gets in the way’.

**Conclusions:**

Tailoring interventions for this population is necessary, and more attention is needed to develop ways to ensure service providers and users engage with behaviour change techniques such as goal setting, rather than focusing on information provision alone. Interventions should also be mindful of cost, cultural diversity, and language and literacy barriers, as well as potential for disengaging this hard to reach population.

## Background

Global rates of obesity have more than doubled since 1980 [1], and obesity is now the second main cause of premature death in Europe [2]. The risk of being overweight or obese is even higher among people of lower socio-economic status [3, 4]. Socio-economic status (SES) is a combined measure of an individual’s income, education level and occupation [5]; essentially those in the lower SES categories typically sit at the lower end of incomes and educational attainment. Other socio-economic indicators exist in England, such as the Indices of Deprivation; a relative measure of deprivation for small areas which also considers measures of crime, health, housing and living environment [6]. Deprivation is multifaceted and measuring SES is complex, but it is gaining increasing interest because of its apparent effect on health and health outcomes [7]. Evidence suggests that the increased risk of obesity in low socio-economic groups is a result of those living in more deprived areas engaging in more unhealthy behaviours (smoking, drinking) and fewer healthy behaviours (physical activity, eat healthy diets) than those living in more affluent areas [8–10]. Adults with lower educational attainment and lower income, both of which are indicative of low SES, have also been found to be more likely to have poorer health literacy [11], and therefore may be less likely to understand the need to lose weight [12].

People from low socio-economic groups not only tend to have poorer health outcomes when compared to people from more affluent backgrounds, but also have poorer outcomes following interventions [13]. Furthermore, changes in government policy to address unhealthy behaviours of the UK population resulted in improvements for those in higher socio-economic groups, yet have been unsuccessful in changing the behaviour of those from lower socio-economic groups [14]. Systematic reviews of health behaviour interventions among low-income populations identified that though lifestyle interventions can be effective for this population, there was a small effect size [15, 16]. White, Adams and Heywood [17] suggested that these poor outcomes following interventions may *increase* health inequalities, highlighting the need to tailor health behaviour interventions for low SES groups. Indeed, evidence suggests that tailoring lifestyle interventions to certain populations or groups can be effective, including diabetes prevention in Latino communities [18], HIV prevention in African American men [19] and smoking cessation in low SES populations [20].

Despite the identified need to tailor lifestyle interventions to low SES populations, a limited evidence base exists to inform their design [16]. A recent qualitative synthesis of overweight people’s experiences of weight management identified psychological and environmental barriers and facilitators [21]. However, only three of the 17 papers included in this review had sampled from low SES populations, and as such, findings are likely too generic to inform the design of such a tailored intervention. Of the previous studies which have focused on low SES populations, the majority have taken place in the USA [22, 23] and the Netherlands [24]. Whilst some issues are likely relevant to low SES groups regardless of location, such as cost considerations [22, 24], it is likely that others will be related to the specific needs of the communities and cultures included, such as American food culture and African American women’s religious settings [22]. Findings from such studies therefore have limited utility in relation to informing the design of weight loss interventions for other populations, for example in the UK.

A further limitation of the current literature is that previous research has failed to consider the views of the intervention deliverers [21, 24]. Including these perspectives is important to understand the challenges faced in delivering interventions to these populations, rather than focusing solely on the challenges of receiving or following such interventions. One previous study identified a dissonance between the views of health professionals, policy makers, and overweight people in relation to their beliefs about causes of, and the most suitable interventions for tackling obesity in the UK [25]. Focusing on issues identified by overweight people alone may result in the design of an intervention which is not considered useful to the deliverer, and therefore may not be implemented as planned. Including both the perspectives of health professionals and overweight people is therefore important to ensure that the design of an intervention for this population is both suitably tailored, and effectively implemented.

### Aims of proposed research

Despite the need to tailor weight loss interventions to low SES populations, limited evidence exists to inform their design. To address this, our study aims to identify important factors to consider when tailoring lifestyle interventions for low SES populations, by drawing from the perspectives of both service providers and service users of a lifestyle behaviour change intervention delivered within a low SES area in the North West of England.

## Methods

### Sampling and recruitment

Participants were identified through a local authority run health improvement service located in the North West of England. This site was selected as the population has poorer health, lower life expectancies and lower rates of employment compared to the UK average (see Table 1).

**Table 1:** Socio-economic parameters for research site, North West and Great Britain

Category	Research site	North West	Great Britain
Long term health problem or disability (2011)			
Day to day activities:			
Limited a lot	11%	10%	8.3%
Limited a little	9.7%	10%	9.3%
Not limited	79.3%	80%	82.4%
Life Expectancy at birth (2007–2009)			
Males (years)	74.7	76.6	78.3
Females (years)	79.6	80.8	82.3
Incapacity benefits (2010)	11%	9%	7%
Employment			
Unemployment rate (2017/18)	4.6%	4.4%	4.3%
All people of working age claiming a key benefit (2016)	14.6%	13.2%	11%

Data taken from Official Labour Market Statistics, www.nomisweb.co.uk, accessed 30/7/18

Participants comprised of staff who facilitate lifestyle change groups (facilitators, F) and members of the public who attend the groups (service users, SU). The group was designed to run for a minimum of nine hours over six weeks, and aimed at any individual living in the area and seeking support around weight control. The free groups were accessed through self or GP-referral, and took place at venues such as health centres, community centres, children’s centres and workplaces. Attendees were weighed each week, and each session covered a different topic, including food diaries and meal planners, behaviour change theory, portion control, the Eatwell guide, food labels, SMART goals, fats and sugars, and the benefits of exercise.

Facilitators were purposively sampled based on their experience in delivering weight loss groups, though they have experience in delivering a large range of health promotion groups within the local authority (e.g. breastfeeding support, cooking courses, physical activity etc.). Service users were recruited through the weight loss groups, both directly by the researcher and by the facilitators delivering the groups. The study was also advertised online, in local community centres and through social media.

### Procedure and materials

We used one-to-one semi-structured interviews to explore participants’ experiences of receiving, delivering and following a weight loss intervention within a low socio-economic community. Field notes from direct observations of weight loss groups were used to inform the topic guides, as well as to increase the validity of the findings from the interviews [26] .

Topic guides were used to organise the semi-structured interviews. Given our inductive approach, topics were broad, but were also informed by both relevant literature [21–24], and observational field notes. Topics covered:

- Views on local areas, facilities available, cost, effect on behaviour change/maintenance.
- Barriers and facilitators to delivering a weight loss programme in the area e.g. exploring the use of goal setting in a group.
- Experiences of following a weight loss programme in the area e.g. which elements of the course were perceived as effective or helpful.

Course material received by the service users during the course were used during the interview as an aide memoire. All interviews were audio-recorded following consent. Service users completed a brief demographic and socio-economic questionnaire which recorded both individual (income, education, occupation) and area (postcode) level indicators of socio-economic status.

### Data analysis

Audio-recordings were transcribed verbatim and analysed following the six stages of thematic analysis outlined by Braun and Clarke [27], which are data familiarisation, coding, searching for themes, reviewing themes, defining and naming themes, and writing up. Data collection and analysis occurred concurrently and iteratively, so that the topic guides continued to be informed by developing themes. This approach also meant that the research team were aware once data saturation had occurred, i.e. when no additional information or themes were being identified in the data [28]. The analysis was undertaken by the first author, facilitated by frequent discussions with the second and third authors throughout the analysis process. The data was coded using NVivo.

## Results

Twenty-five participants were interviewed (11 F, 14 SUs). Mean length of the interviews was 49 min (28–78), and took place face-to-face in people’s homes or places of work. One SU interview was conducted over the phone due to ill health. All facilitators worked in health promotion within a local authority service providing free groups or one-to-one support in the community. Facilitators covered 19 distinct areas, with a wide range of IMD deciles (1–10).

Of the 14 SUs, 13 were female. The majority of the sample was White British, with just one Asian women. Their mean age was 66 years (44–84). Individual IMD deciles based on SUs postcodes ranged from 1 to 10^1^ (mean 4). Though this is a broad range, 10 of the ratings were 3 or below, and represents the diversity of the area. Two SUs were in employment, 10 were retired and 2 were unemployed. Eight SUs had qualifications, with the highest being degree level (Table 1).

**Fig. 1:**
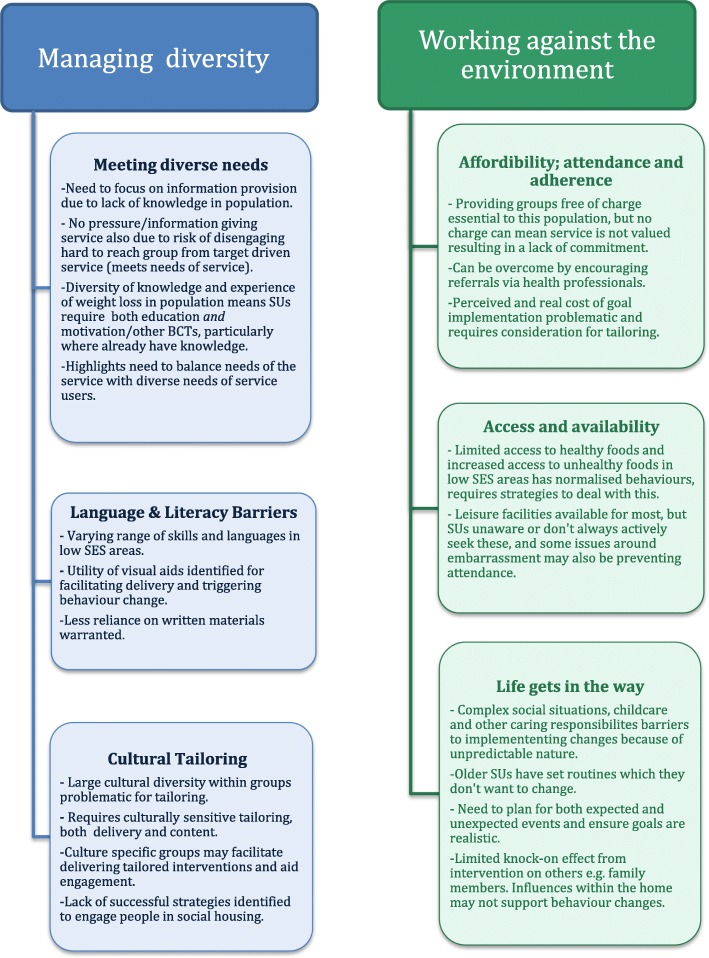
Summary of findings across the two main themes. Both themes, *Managing diversity* and *Working against the environment,* with a summary of their three subthemes

We identified two broad themes relevant to tailoring the delivery and content of interventions across the interview and observation data; 1) Managing diversity and 2) Working against the environment. These themes and their subthemes are discussed below, and are presented in Fig. 1.

1. **Managing diversity**

**Table 2:** Service User characteristics

ID number	Age range	Occupation	Ethnicity	Highest qualifications	IMD decile^1^
SU1	75–79	Sales and customer service (Retired)	White British	None	9
SU2	45–49	Skilled Trades	White British	NVQ level 3	3
SU3	60–64	Administrative and Secretarial (Retired)	White British	NVQ level 2	3
SU4	65–69	Administrative and Secretarial (Retired)	White British	Vocational	9
SU5	70–74	Administrative and Secretarial (Retired)	White British	1 A level	1
SU6	80–84	Elementary (Retired)	White British	None	1
SU7	70–74	Elementary (Retired)	White British	None	3
SU8	65–69	Caring, leisure and other (Retired)	White British	Level 2 diploma	10
SU9	60–64	Unemployed (Employment Support allowance)	White British	None	2
SU10	70–74	Elementary (Retired)	White British	Degree	2
SU11	55–59	Caring, leisure and other	Asian British	Level 2	3
SU12	65–69	Elementary (Retired)	White British	Vocational	6
SU13	40–44	Unemployed (Employment Support allowance)	White British	None	1
SU14	65–69	Manager (Retired)	White British	None	1

We identified that the diversity of knowledge and experience of healthy living, language and literacy skills, and cultures among this population presented challenges both in the delivery of, and engagement with, a generic group intervention. Some challenges were overcome by facilitators, and are highlighted as important lessons for tailoring interventions for this population in relation to facilitating intervention delivery and behaviour change.

### Meeting diverse needs

We identified that knowledge around healthy living varied widely within the target population. Facilitators identified the lack of such knowledge as the main barrier to behaviour change in this population, and felt therefore that there was a need to focus the intervention on educating service users around healthy lifestyles:

*I think it’s like down to education, because they’ll get people say ‘I had a meat and potato pie, but I only had one. That’s good, isn’t it?’ And it’s like, well, actually, no, it’s a meat and potato pie… when we had broccoli, and it’s like, ‘Is that a green cauliflower?’ And it’s like, oh, my gosh!...So it’s like starting from scratch. (F7)*

The lack of knowledge around healthy eating in particular was supported by our observations where attendees asked questions such as if crisps contribute towards their ‘five a day’ (Obs5), and if ice-cream and chocolate are categorised as dairy products or fat in relation to the eat-well plate (Obs3).

As well as the identified need for education, the facilitator’s preference to focus on information provision may also reflect their preference to run *“a no pressure group”* (F1) for this population. For example, facilitators described the use of tools such as food diaries and goal setting among service users as *“a bit hit and miss” (*F2), and generally did not apply any pressure on service users to engage with the materials because of the risk of disengaging this hard to reach group:

*They’re coming voluntarily, and if you start really, sort of, preaching to them, they’re not going to come back anyway…(F4)*

Similarly, facilitators expressed concern about using weight as an outcome measure for service users because of the risk of poor outcomes demotivating and ultimately disengaging people from the intervention:

*I think because so many people do have that perception of, like, two to three pounds is the only way that I can be successful, I mean, a lot of the time people, you know, if they have only lost half a pound, they can still feel downhearted and it will ruin their confidence to carry on going. (F6)*

Therefore, as well as balancing the perceived needs of the service users around lack of education, the ‘no pressure’ information provision approach also met the needs of the *“target driven service”* (F1) in terms of maintaining engagement and keeping *“bums in seats”* (F1).

Though some SUs reported liking the no-pressure approach as they felt comfortable, this approach didn’t necessarily encourage people *“to obligate or commit themselves in any way”* (F1) to promote change. Indeed, we observed that many of the service users interviewed had not used the tools in the folders provided, other than the weekly weigh in record which had been completed by the facilitator. One SU in particular identified the lack of pressure as a reason for this, suggesting that more pressure may be beneficial to engagement with the tools:

*I: Do you know why you didn’t use them [food diary, meal planner]?*

*SU8: Lack of incentive I would say…I think I would have found it more useful if I was being checked up on, I know that sounds pathetic, doesn’t it? But if we had to fill in a sheet and could, sort of, hand it in every week and whoever was taking the class could just have a glance over it and see where you’re making mistakes and just encourage you to change certain things, I think that would be better.*

Indeed, contradicting the need to focus on education as suggested by facilitators and our observations, the majority of the SUs we interviewed had previously attended weight loss groups and felt they knew most of what was being taught. Rather than a need for education, these SUs were attending mostly for the social support/pressure and motivation to make the changes required to lose weight:

*I mean the thing is, I know what I should do, I know what I should be doing, I know what I should have, I just don’t do it, you know. But when you’re going and being weighed every week and it has helped I think. (SU5)*

Also in contrast to facilitator’s concerns, service users clearly valued the weekly weigh in as a useful behaviour change tool. Indeed we observed some service users who turned up only to be weighed and left immediately after, supporting that some perhaps didn’t value the educational and social aspect of the groups. Many service users put past weight loss success down to having weight monitored by a health professional or a weight loss group, and their subsequent weight regain highlights the importance of on-going monitoring for weight loss maintenance:

*When I’ve lost the most weight was when actually it was the doctor mentioned about my weight and decided that I would go into the doctors every fortnight to be weighed and that was a very good incentive… because I was having to go back to the doctors regularly to be weighed, I lost three stone, but then when you stop that, it creeps back up. (SU8)*

### Language and literacy barriers

Another main challenge in the most deprived areas in terms of intervention delivery was that those attending the groups had *“varying skills”* (F4) in terms of language and literacy abilities. This was due to both an influx of refugees, asylum seekers and immigrants, and poor literacy levels among those originally from the area:

*I: And has the change that you mentioned, in the area, has that impacted your work in any other way?*

*F7: I just think perhaps working with most people, a lot of people that are asylum seekers and refugees, so the language barrier is quite difficult…because English isn’t the first language, that’s really difficult for me to give them leaflets and understand. But also, if English is the first language, the literacy in this area isn’t…I think it’s an average age of nine or something.*

Language and literacy barriers were particularly problematic given the focus of the intervention on educating service users, and facilitators identified that educating people on reading food labels was particularly challenging:

*I’d say label reading is probably one of the harder ones, especially if people have, sort of, literacy issues or anything like that …especially with the writing on labels, a lot of the time it’s so small and they’re all labelled completely differently, so some will say Sodium, some will say Salt and, you know, those types of things, I think that’s really difficult. (F6).*

The diverse population and broad range of education also meant that there were also many who had no problems with language or literacy. This provided a challenge in terms of how information was presented to the groups to suit all:

*We had our booklet and whichever page they were dealing with like the fats and the sugars and things it was kind of like they just read it to you. I mean, I’m not stupid and I’m quite clever and to be just read to. I can read to my children, you know, story time and things when they were younger. (SU2).*

One way facilitators overcame this barrier was through using visual aids, such as pots of fats, sugar and salt contained in commonly consumed foods and drinks. Rather than relying on printed materials, these resources were valued by the facilitators and service users alike:

*I: Ok. Are there any elements of the course that are particularly successful, or any elements that the group uses a lot?*

*F2: Yeah, I think having examples, so the sugar pots that have how much sugar is in a bar of chocolate or fizzy drinks and that kind of thing… so they are quite effective at you know, showing people what you’re talking about when you’re talking about grams of sugar or teaspoons of sugar, to see what it actually looks like you know.*

These visual aids were regarded by facilitators as *“massively important”* (F7) for overcoming language barriers, and were also valued by service users as important behaviour change triggers:

*I’ve not bought a sausage roll, because I’ve seen all the fat that’s in it, so actually seeing the stuff that’s in food, I think is good… I don’t think I’ve had more than one pie in the last six weeks because I’ve seen how much fat’s in the pastries and that. Sausage rolls, they disgust me. Sausages, bacon’s full of salt, and I’ve never…I’ve bought one packet of bacon since I’ve been coming and it’s still in my fridge. (SU4).*

Facilitators also distributed wallet sized food label cards which focused on the visual traffic light system to aid SUs with making healthier choices when food shopping. Many SUs valued these and reported using them:

*When I go shopping, I get my little card out and have a look, whether they are green or orange or red... it’s surprising what’s in things I think, so it’s educated me on that, definitely. SU4.*

Not only did language and literacy levels raise issues regarding presenting information in a suitable format, but it was also identified as another barrier to people engaging with the materials, tools (e.g. food diaries, goal setting) and activities used as part of the course. Facilitators expressed that getting people to write anything down was *“a massive chore”* (F12). Although it was unclear if this was due to literacy levels, or other reasons such as motivation, difficulties with reading and writing were observed in three of the five observations, and resulted in those SUs not being able to fully participate with the group activities (following a workbook, identifying the number of calories in party food, goal setting). Facilitators identified that in the *“majority of cases”* (F4), SUs were also unable to set realistic goals following simple instructions, something which was also observed in our observations. Given the time constraints, many facilitators could not assist individuals with this task, leaving many with unrealistic or vague goals.

### Cultural tailoring

Facilitators acknowledged that each session they ran required tailoring, given the diversity of the attendees and the number of different behaviours that need targeting:

*You could have a group of 10 clients, and you’d do the alcohol session, and for 3 or 4 of them it won’t mean anything because they don’t really drink, and equally you might do the session about fats and sugars, and it might not mean anything to anybody because that person doesn’t really eat cakes of biscuits. (F1).*

*You have to tailor [the intervention], although you have got the programme you have got to make the programme fit the people that you have got coming to it. (F9).*

Adding to the complexity of tailoring, analysis identified large cultural diversity in a relatively small geographical area, which was highlighted as challenging in relation to delivering a generic group intervention to a range of needs:

*[Area name] is much more diverse in terms of people moving into the area, so other cultures moving into the area, so there are quite a lot of Eastern Europeans here, quite a lot of African families, erm, and they bring all different needs with them as well, so the two areas, even though geographically they’re very, very close, they’re quite different, and what groups works in [other area] may not work in this area because the client base can be so, so different, so it’s quite a juggling act really.(F1).*

Facilitators identified that people originally from the area tended to have poor diets in terms of the amount of fresh food and number of take aways and ready meals consumed. In contrast, those who had moved into the areas from outside the UK, such as refugees and asylum seekers, reported eating lots of fresh, and limited processed foods, but cooked the food in an unhealthy way:

*But as far as weight support, I think they’re at different levels, because their diets are different and their cultures are different. So if you’ve got a diverse group, it is quite difficult because they’ll find…I’ll say, who goes to McDonalds? And say, all [research site] born people go, I go and I take the kids. But the ones, like from Ethiopia or Somalia, will go, hmm, I never go to McDonalds. So their diets aren’t anywhere near as bad as ours. It’s just purely down to how they cook their food. (F7).*

Facilitators therefore needed to target different behaviours and provide different information for the different types of attendees within one group. This also relied on facilitators having sufficient knowledge around what is culturally appropriate, which was not always the case:

*We worked through Ramadan and that was difficult, you know, because you don’t eat during the day and you only eat during the night and it’s...that was difficult. I mean, some of them lost weight, but even adapting their food to, you know, a healthier lifestyle was difficult, because I wasn’t sure of what they ate. I mean, they cook from scratch, but they use...they always use fresh ingredients. So, you weren’t looking at food labels, you were just looking at the amount of fat and stuff that they put in. (F9).*

One approach adopted to manage cultural diversity in some areas was to have separate groups targeting different communities. For example, the service ran a Jewish women only weight loss and exercise group, a weight loss group for ethnic minority women and an asylum seekers and refugees weight loss group. These groups tended to be well attended, and facilitators felt that this success was due to the amount of time and effort put into the initial relationship building, ensuring cultural sensitivity (such as dressing appropriately, tailoring the content), choosing appropriate venues, and subsequent word of mouth within the communities.

*They don’t go to GPs and they don’t access any services that aren’t Jewish specific, so to speak. So when I went, I tended to dress sensitively to them as well. And then they tended to accept me, and then a lot of the ladies then passed round that there was this weight support programme and it worked really well in the Jewish community. (F7).*

Although this approach had been successful with running groups who shared certain ethnicities or religion, it had not been very successful with people *“born and bred”* (F7) in the area, such as with young mums or working age people living within the most deprived areas. Facilitators had targeted these specific communities and had ran groups within social housing or in pubs rather than their usual community venues, but described these communities as *“a bit closed doors on you”* (F2), and as numbers dwindled such groups did not continue. This suggests a different approach may be required for engaging such hard to reach groups, though we identified no alternative approaches in the data.

2) **Working against the environment**

External environmental issues were identified as challenges to both the delivery of the intervention, as well as barriers to users achieving their lifestyle behaviour goals. Giving consideration to such factors is important with regards to designing interventions with which this population can realistically engage.

### Affordability; attendance and adherence

Running groups free of charge was highlighted as important by facilitators and *“crucial to the area”* (F3) because of the level of deprivation, as it made the groups available to all. Similarly, service users identified that *“you don’t have to pay”* (SU5) as a reason for selecting the course above others in the area. Despite this, facilitators were concerned that being free meant that people may not value the course, preferring instead to opt for expensive commercial weight loss groups, despite the cost:

*People think if it’s free might be nothing, they’re not interested. They go…in this building, Tuesday afternoon they have a big Slimming World session, big Slimming World session. And I had a [weight loss group name] programme, just eight. (F10).*

The lack of value of a free course was overcome in one area by promoting the groups with local GP practices, such that GPs could refer patients to the group:

*… they just think it’s just a free course, it’s not really worth anything, erm, but yeah, I think trying to get more, working more closely with the GPs and going down that route has been a bit more effective in getting people in that way… you know, if they’ve been told to go by a GP or practice person they put a bit more weight behind it. (F2).*

Facilitators also identified that the lack of a financial investment by service users could affect longer term commitment to the course for some:

*If you said that, you know it’s forty quid for the next 6 weeks, if they paid that upfront, are they more likely to go because they don’t want to waste the money? Whereas because we’re very low pressure, very informal, it’s all free, you can come if you want, don’t come if you don’t want, whether people don’t place any value in that, and they think well I didn’t pay for it so I’m not losing anything, I don’t know, it’s a tough one. (F1).*

However, facilitators felt the course must remain free to ensure access for all, and no suggestions were made for improving long term commitment in the absence of financial investment.

As well as costs relating to attending the course, costs associated with implementing behaviour change were also highlighted. Though the facilitators in this study were clearly aware of cost issues and tailoring information and recipes to the service users’ budgets, one service user highlighted the importance of ensuring the intervention content is tailored to the population in relation to cost, giving an example from a different weight loss group running in the area:

*…in the group, there’s a lady and she’s got 4 children, and she’s put on weight…and they’re talking about, if you like a burger, go buy a nice piece of steak and mince it, and she said “I can’t afford steak, I’ve got 4 kids and limited amount of money, I just can’t afford it, I have to buy the kids cheap burgers”. She lives in [area], a really deprived area, and the lifestyle coaches don’t have an answer. (SU14).*

As well as the real cost attached to eating healthily, facilitators shared a frustration around the local population’s perceptions around the cost of eating healthily, with most expressing that eating healthily *“costs a fortune”* (F3):

*…the actual people of [city], tend to be obsessed that fruit and vegetables are just too expensive. And they would rather…the kids may have like a fruit juice drink or a Greggs pasty but the cost of them… But it’s just something’s set, that everyone goes, ‘oh no, fruit and veg, really expensive. I can’t afford to eat healthily.’ So it’s about changing…probably more education as well. And changing the way they think, which is quite difficult. (F7).*

Facilitators tended to offer solutions to this, such as providing cheap recipes and signposting to local supermarkets which have special offer fruit and vegetables. Those with limited cooking skills or confidence were also signposted to free, local cooking groups.

Service users were provided with a leisure pass as part of the course, allowing them free access to local gyms. Facilitators and service users felt these were beneficial because many in the area couldn’t afford to pay for gym memberships, and so they could still increase their exercise levels without worrying about cost:

*I: D’you think you’d go otherwise if [F2] didn’t give you the pass? D’you think you still would have gone [to the gym]?*

*SU4: Well, I’d definitely go to the swimming. I don’t know about the gym. I think the gym’s quite expensive, ‘cause I only get a pension. You know, so...*

However, there was a mixed response in terms of their use. Whilst some service users valued them and used them throughout the course, others were not motivated to use them, and found there were other barriers to physical activity, such as time and motivation, or simply that they don’t want to, or don’t have the confidence to attend the gym on their own:

*They’re quite happy to come out and go on the walk every week with me, but the minute I say ‘there’s your gym pass, go yourself’, they don’t go. So that’s confidence as well. And to try to overcome that, I have gone with them and said like, could they buddy up and go with somebody else, and some have done it and worked really well, but as a rule, I don’t think a lot use the passes.* (F7).

### Access and availability

Not only were there issues around cost in relation to buying healthy food, but its availability in the area was also identified as an issue to making dietary changes. The most deprived areas were highlighted as having limited availability of fresh and cheap healthy food, and many service users described having to take at least one bus to get to their nearest supermarket, which meant sometimes taking a taxi home because of the weight of the shopping.

*F9:…There’s an Aldi and a Lidl. So there is a choice for them.*

*I: Is that easy to get to?*

*F9: Yeah, it’s only, well there is a bus and you find that some of them will get taxis. But it is sad that there’s not an Aldi on here for them. ‘Cause they’re sort of left on their own, aren’t they, this side of the dual carriageway, and there’s nothing here.*

*SU10: We’ve hardly got any shops here. There’s the Post Office and then there’s the chemist, next door to that is [sandwich shop], and then it’s a do it yourself shop, that’s it. If you want anything we have to go to Morrisons, sometimes you see I go down on the bus to Morrisons and then hopefully if it’s the one where I’m not buying such a lot….*

In contrast, the availability and convenience of fast food was highlighted as a particular problem in the most deprived areas by both facilitators and service users alike, given the constant temptation to eat unhealthily. Indeed, alongside portion control and lack of physical activity, facilitators identified frequency of fast food meals consumed as one of the main contributing factors to weight gain in the area.

*In [area], I think that there are too many takeaways, because nearly every other shop is food, so there’s loads of it, it’s just so convenient, you know, some areas don’t have as many as this, so you tend to find that they won’t eat it as much, because it’s not there, but in [other area], there are a lot of takeaway places and more opening up. (F5).*

*There are an awful lot of takeaway places in both areas…They’re both full of takeaways and pubs which seems to describe both areas quite well…the access to the takeaways is very…it’s just so easy in both of these areas because they’re just within moments of walking... Whereas when I go and stay with, well, I go to my brothers’ houses, they’re in a more affluent area and there’s hardly any takeaways near where they live. (SU2).*

The physical availability of convenience foods also meant that its consumption had been normalised, and the social acceptability of living unhealthily in the area was highlighted as a barrier to behaviour change:

*I think the general culture within [city] can sometimes make it really difficult for people to lose weight, so there’s lots of convenience food, there’s lots of takeaways, it’s very normal for people to eat takeaway food, convenience food, you know, it’s very much the norm. (F6).*

Facilitators tailored the groups in response by suggesting buying tinned and frozen fruit and vegetables instead of fresh to avoid having to go to the shops as often, as well as providing recipes for cooking healthier versions of popular take aways, such as chips.

Unlike access to healthy food, few issues were identified with access and availability of leisure facilities in the area. Facilitators and SUs reported lots of free leisure activities happening on a regular basis, such as free walking and cycling groups:

*… there’s plenty going on, you know, there’s, if you look for places you know, look for things to do, there’s plenty to do that cater for everybody really… If you look for things to do you know, you can always find something, I mean I’ve been swimming this morning (SU1).*

In contrast to the gym, these more social type groups were reported to be well attended, particularly by the over 50s. Rather than issues with accessibility, the only issues we identified were SUs simply being aware what was available in the area, or issues around being body conscious:

*Well erm, there’s [name] country park at the back of here, they do a few things there, but I’m not sure what they are…I wanna go see what they do at the [name] park, I do believe that on a certain day, I dunno when it is, that they provide bikes and walking (SU13).*

*I enjoyed swimming, I swum for donkeys’ years, erm, mainly its vanity. You know, you don’t want a fat old bloke jumping in the pool (SU14).*

### “Life just gets in the way”

One common reason given for why people didn’t follow the intervention or adhere to their goals was that people in the area simply have too many other things going on in their lives to allow them to prioritise their health and their weight loss. Issues such as complex social situations, childcare and caring responsibilities were all identified as particular problems as to why service users either did not continue to attend, or didn’t achieve meaningful outcomes, despite their best intentions:

*The people who do attend, I just find that a lot of the time, they’ve just got a lot of other things they’re trying to deal with, …childcare can sometimes be an issue… a lot of people have a lot of other things that are going on in their life that they need to prioritise over weight.…it’s just life, I just think life just gets in the way for people when they’ve got other things that they prioritise and then all of a sudden, the thing that they really wanted to do is just at the back of their mind and it just gets swallowed up, I’d say that’s probably what happens the majority of the time. (F6).*

Facilitators recommended meal planning for the week as a way to help service users eat more healthily, but given their busy, complex lives, some found this problematic. For example in our observations one person highlighted that their caring responsibilities and its unpredictable nature meant that achieving their goals (planning meals and exercise) that week had not been possible (Obs5). This particular individual had planned to defrost a healthy meal in the morning ready for the evening meal, but there had been an emergency health issue, and the family had ended up eating unhealthily in the evening, which had been a common occurrence.

Given that many of those were interviewed in our study were retired, time was not identified as a barrier to change, but being older meant that they had very established routines which were difficult to break, especially routines around eating out and having treats:

*They can be quite set in their ways, they have their certain routines that they like to stick to, the like to go out for meal if they’re retired, they like to spend their free time doing those kind of things. (F2).*

One way this was managed by facilitators was by trying to highlight that “*life’s not going to stop because you want to lose weight*” (F6), and to focus on providing damage limitation strategies in particular situations, such as choosing the healthiest options when eating out or attending parties. Though useful for planned events such as going out for lunch, we identified no strategies for helping people adhere to their goals when also dealing with unplanned situations. Rather, facilitators felt that if no changes were being made, then it was perhaps simply not the right time for the individual to be taking part in the intervention.

We identified unhealthy culture within the home environment as a barrier to behaviour change, particularly in relation to the influence of partners and spouses:

*I’d say there’s a few that’ve been on the same kind of diet for years, or if they’re married some of them struggle with trying to get their partners on board as well, it’s a difficult thing if I say you need to reduce your saturated fat content or sugar content, they’ll say ‘well my partner’s not going to be very happy’, and then trying to negotiate with that and trying to get them to make that change. (F2).*

One service user identified that they had since changed the contents of their children’s lunchboxes for healthier alternatives as a result of their own changes made following the course (SU2). However, many discussed the temptation of having ‘treats’ in the house for children and grandchildren, suggesting that there had been no ‘knock-on’ effect on the family:

*The only thing that I should really be getting is like, me lads aren’t here now, I can’t deprive them of all the snacks and stuff, but obviously I eat them, and I, do you know what I mean? (SU13)*

*I: Anything that you found difficult or challenging about losing weight?*

*SU11: Giving the grandchildren treats and things and not being able to have them. That was difficult, saying no to yourself.*

This suggests that changes made as a result of the interventions didn’t seem to affect attitudes, or behaviours beyond the individual level. This lack of impact and lack of support at home has implications for supporting the longer term behaviour of the individual, and therefore may need addressing as part of the intervention, though we identified no strategies in the data.

## Discussion

### Summary of main findings

Our results highlight important challenges and facilitators to delivering lifestyle interventions to low SES populations, as well as suggestions for how such interventions may be best tailored. Firstly, three subthemes fit within the overall theme of *Managing diversity*, which highlights the heterogeneity of low SES groups. Facilitators identified that the focus of an intervention for this population should be on information provision to address the lack of knowledge around healthy living. The no-pressure approach met the needs of the service, ensuring that they didn’t apply too much pressure resulting in disengaging this hard to reach group. However, many service users felt that they had the necessary knowledge, but were seeking support and monitoring to help them make lifestyle changes, suggesting that more pressure was acceptable and perhaps necessary to achieve meaningful outcomes. Diversity of language and literacy skills meant that delivering the course was challenging, but visual aids facilitated the delivery of the educational aspect of the intervention, and also triggered behaviour change among service users. We did not identify any strategies to encourage engagement with the intervention tools however. Cultural diversity was also an issue in terms of tailoring the content of the course, and highlighted the importance of ensuring that the intervention deliverer has an appropriate level of knowledge of different cultures in the area. Targeting certain ethnic and religious groups had proved successful in terms of engagement, primarily due to tailoring content and relationship building. However, similar efforts to target some communities such as social housing had not been successful in the past.

The second overarching theme *Working against the environment* incorporated all issues related to the environment, including individual resources, such as the cost of attending a course and of implementing the changes. Providing the course for free was identified as one way to ensure equal access and encourage engagement, but may have hindered commitment. Access and availability of healthy and unhealthy foods was also prominent in terms of poor access resulting in poor food choices. The availability of community resources such as leisure facilities and green spaces in the area was identified as good and plenty of activities were available, but were varied in their use. Many of these environmental challenges were barriers to SUs implementing their goals, and highlights the need for intervention content to be mindful of both cost and ease of goal implementation. Finally, although strategies were used to help people with planned events (e.g.as eating healthily when out), some service users found achieving their goals difficult because of the unpredictable nature of their complex lives. The influence of family members and the normalisation of unhealthy lifestyles at home were identified as barriers to behaviour change, particularly in the long term.

Both themes identified some ways in which lifestyle interventions can be tailored to low SES populations in relation to facilitating delivery and supporting behaviour change. A summary of these recommendations can be seen in Table 2.

### Relevance of findings

As previously discussed, people living in low SES areas tend to benefit less from interventions compared to those from more affluent backgrounds [13], resulting in an increase in health inequalities. The issue highlighted in our results regarding the focus on a low-pressure, information giving service because of the fear of disengaging a hard to reach group may be a contributing factor. Whilst information provision is an important element to include in an intervention, it is unlikely to change behaviour on its own [29]. Furthermore, a recurring issue in the themes was that engagement with behaviour change techniques such as goal setting, food diaries and meal planning was poor, due to a combination of language and literacy barriers, no pressure or incentive to complete them, and the complex lives of those attending the groups. As such, strategies are required to ensure both service providers and service users in low SES areas engage with other behaviour change techniques known to be effective for weight loss, such as goal setting, self-monitoring and goal review [30, 31]. Given the complexity of people’s lives and language and literacy barriers, low SES groups may need more support in setting of their goals, to ensure that their goals are recorded and easy to implement. Our results also suggest a need for strategies to increase commitment, but also to balance this with maintaining engagement with the service.

One particularly important and relevant subtheme identified was the literacy and language barriers which highlighted the importance of using an appropriate delivery method for interventions delivered to low SES populations, such as the use of visual aids. A recent editorial identified the form of delivery as an ‘active ingredient’ in the delivery of behaviour change [32] and may be particularly important when considering the language and literacy abilities of the end users.

Previous research supports our findings in relation to barriers such as perceptions of, and actual cost of living healthily among low SES populations [21, 24, 33]. Interventions for this population should ensure costs are considered in relation to recommendations made.

The majority of evidence supports our findings in relation to access and availability of healthy and unhealthy foods. In the US, accessibility or availability of healthier food and limited access to convenience food is associated with better dietary outcomes and lower levels of obesity [33, 34]. UK based studies have identified that distance from shops affects diet alongside other social and economic factors [35], and that increased exposure to take away outlets is associated with increased consumption of fast food and increased body mass index [36]. This suggests that in the UK at least, a whole systems approach to tackling obesity is required, targeting both individual level factors through interventions, and environmental factors with changes in policy in relation to availability of healthy and unhealthy foods. Indeed, adding a sugar tax to soft drinks in the UK is one such recent approach to tackle obesity on a large scale. Such approaches have shown promise elsewhere [37], and relevant to our population, modelling has suggested its effectiveness across income groups in the UK [38].

We identified that in the main, service users in our study did not change the diets of those around them, and were sometimes not supported to make their own changes at home. Similarly, a previous synthesis of experiences of weight management concluded that the majority of their 17 studies identified family and friends as a barrier [21]. In a recent systematic review of grandparents’ influence on children’s cancer risk, grandparents were identified as “indulgent”(p28), and were found to have an adverse effect on weight, diet and physical activity [39]. This supports our findings given that many of our service users were parents or grandparents, and discussed the difficulty of resisting treats they had bought for their families. This suggests that lifestyle interventions needs to consider how best to involve, or effectively share messages with the entire family to both support individuals’ long-term changes, and to begin to tackle the normalised unhealthy culture within the home environment.

What this study tells us is how interventions might be tailored to improve intervention engagement and outcomes for people who access services, but not how to improve engagement with those who don’t or are not motivated to seek help. By nature these people are difficult to access, but in order to identify what might motivate them to engage, more research is needed with these particular sub-groups. Similar work has been conducted in relation to engaging low SES populations in chronic heart disease primary prevention [40]. The authors identified that engagement strategies must also be tailored to the population, particularly given the hard-to-reach nature of low SES populations.

### Strengths and limitations

One of the strengths of this study is that views were obtained from both the service user and service provider perspectives, as well as supported by direct observations. This is particularly important given that their views sometimes differed, and that previous research has focused on service users only [21, 24]. For example, a single lens approach focusing on the views of service users only would not have captured the need for education, nor the challenges faced in delivering the intervention to such diverse groups. Similarly, solely using staff views would have mainly suggested a need to focus on education and not on the need to increase pressure/ engagement with the tools. Observations also strengthened the views collected by interview, and helped identify that both views were valid by highlighting the diverse nature of those who attended the groups.

Another strength is that our study took place in a real world setting in a city which comprises of both deprived and affluent areas. Participants were from different teams and from a range of socio-economic areas across the city, which provided variability within the sample (see Table 3). Though it was set within one service we do not feel this limits the findings as the delivery and content of the course varied widely from group to group. Given that the service was a local authority run service, they were more likely to use evidence based/government recommendations than some more commercial groups. As such, findings from this study may be more relevant to intervention developers and practitioners than if we had included commercial services.

**Table 3:** Challenges identified and suggested tailoring for lifestyle interventions for socio-economically deprived populations

	Themes identified	Suggestions for tailoring (data)	Further suggestions for tailoring
Managing diversity	Meeting diverse needs	• Focus on education and no pressure to engage with tools for those with limited knowledge and difficult to engage.	• Separate groups for first time attendees with focus on education, and then on-going weigh-in and support groups for those who have previously attended.
	Language and literacy barriers	• Visual aids e.g. fats, sugars and salt pots, traffic light card.	• More visually presented information rather than reliance on written materials.
	Cultural diversity	• Target specific groups e.g. ethnicity, religion, to allow for tailoring of content and building relationships.	• More community development and linking with social housing. • Ensure service deliverers are suitably trained to deliver culturally sensitive information.
Working against the environment	Affordability; attendance and adherence	• Use health professional referrals to add value to free course. • Provide cost appropriate suggestions e.g. local deals, cheap recipes. • Linking with leisure facilities for special offers.	• Additional commitment element to course. • Considerations for policy level e.g. food vouchers.
	Access and availability	• Recommend frozen and tinned fruit and vegetables. • Suggest best options for fast food e.g. tomato rather than cream based curries. • Signposting. • Free leisure pass.	• Consideration for policy level e.g. planning. • Include strategies for replacing fast food e.g. cooking own healthier versions. • Interagency communication to identify gaps in provision.
	Life gets in the way	• Planning meals. • Damage limitation strategies e.g. knowing what not to eat at parties.	• Ensure easy to implement/ realistic goals. • Strategies to encouraging partners and families to support/ adopt changes.

A limitation of our study is that all of the service users involved were white British, with the exception of just one Asian woman. Though this mainly older female population represents those who attended the groups, results may not be fully representative of the area, particularly given the reference to cultural diversity. We feel that including interviews with both staff and service users, as well as direct observations gave a more rounded view of the needs of all service users who used the service, not just those willing to participate in our study. However, it is likely that there is still a lack of transferability to designing interventions to specific low socio-economic cultural groups. For example, in a synthesis of barriers to physical activity in South Asian adults, issues specific to the culture were identified, such as language and group norms [41]. Though some of these issues were touched upon in this study, further research specifically with these sub-groups would be required to appropriately tailor such interventions.

We also had only one male in our study, which reflects the disproportionate number of males accessing these types of services. Gender is an important consideration in terms of engagement with services, with the under representation of males in such services previously identified as an issue [42, 43]. Addressing this, Football Fans in Training (FFIT) is an intervention run in professional football clubs, which is gender sensitised in terms of content, context and delivery style [44]. Not only has FFIT shown that tailoring an intervention to males in this way is an effective strategy in terms of engagement [45] and weight loss outcomes, it was also effective across socio-economic groups [44]. This suggests that when targeting males, tailoring the intervention based on socio-economic groups may not always be necessary when tailoring in other ways.

## Conclusion

Results suggest that tailoring lifestyle interventions for socio-economically deprived populations is necessary given the specific challenges identified. Such interventions should not rely solely on information provision; particularly written material should be avoided where possible given the language and literacy barriers identified, and replaced with more visual resources. Intervention content should be mindful of both cultural diversity and cost of recommendations. More attention is needed to develop ways to ensure service providers and users engage with effective behaviour change techniques, such as goal setting, ensuring these are realistic and achievable. Intervention designers should be mindful of balancing increased commitment with continued engagement for this hard to reach population.

## Abbreviations

F: Facilitators
SES: Socio-economic Status
SUs: Service Users

## Footnotes

1 Rating 1–10, where 1 is in the 10% most deprived areas in England.
