# Correction to: Tailoring lifestyle interventions to low socio-economic populations: a qualitative study

**DOI:** 10.1186/s12889-018-5979-3

**Published:** 2018-09-28

**Authors:** Nia Coupe, Sarah Cotterill, Sarah Peters

**Affiliations:** 1 Manchester Centre for Health Psychology, School of Health Sciences, The University of Manchester, Manchester, UK; 2 Centre for Biostatistics, School of Health Sciences, The University of Manchester, Manchester, UK

## Correction to: BMC Public Health (2018) 18: 967. https://doi.org/https://doi.org/https://doi.org/10.1186/s12889-018-5877-8

In the original publication of this article [1] there is an error in the citations of Tables 2 and 3. In this correction article the incorrect and correct citations are shown for clarity:

Incorrect:

Eight SUs had qualifications, with the highest being degree level (Table 1).

Another strength is that our study took place in a real world setting in a city which comprises of both deprived and affluent areas. Participants were from different teams and from a range of socio-economic areas across the city, which provided variability within the sample (see Table 3).

Both themes identified some ways in which lifestyle in terventions can be tailored to low SES populations in relation to facilitating delivery and supporting behavior change. A summary of these recommendations can be seen in Table 2.

Correct:

Eight SUs had qualifications, with the highest being degree level (Table 2).

Another strength is that our study took place in a real world setting in a city which comprises of both deprived and affluent areas. Participants were from different teams and from a range of socio-economic areas across the city, which provided variability within the sample (see Table 2).

Both themes identified some ways in which lifestyle interventions can be tailored to low SES populations in relation to facilitating delivery and supporting behaviour change. A summary of these recommendations can be seen in Table 3.

Tables 2 and 3 are also shown in the Correction article for reference. The original publication has been updated. The publisher apologizes to the authors and readers for the inconvenience.

**Table 2:** Service User characteristics

ID number	Age range	Occupation	Ethnicity	Highest qualifications	IMD decile^1^
SU1	75–79	Sales and customer service (Retired)	White British	None	9
SU2	45–49	Skilled Trades	White British	NVQ level 3	3
SU3	60–64	Administrative and Secretarial (Retired)	White British	NVQ level 2	3
SU4	65–69	Administrative and Secretarial (Retired)	White British	Vocational	9
SU5	70–74	Administrative and Secretarial (Retired)	White British	1 A level	1
SU6	80–84	Elementary (Retired)	White British	None	1
SU7	70–74	Elementary (Retired)	White British	None	3
SU8	65–69	Caring, leisure and other (Retired)	White British	Level 2 diploma	10
SU9	60–64	Unemployed (Employment Support allowance)	White British	None	2
SU10	70–74	Elementary (Retired)	White British	Degree	2
SU11	55–59	Caring, leisure and other	Asian British	Level 2	3
SU12	65–69	Elementary (Retired)	White British	Vocational	6
SU13	40–44	Unemployed (Employment Support allowance)	White British	None	1
SU14	65–69	Manager (Retired)	White British	None	1

**Table 3:** Challenges identified and suggested tailoring for lifestyle interventions for socio-economically deprived populations

	Themes identified	Suggestions for tailoring (data)	Further suggestions for tailoring
Managing diversity	Meeting diverse needs	• Focus on education and no pressure to engage with tools for those with limited knowledge and difficult to engage.	• Separate groups for first time attendees with focus on education, and then on-going weigh-in and support groups for those who have previously attended.
	Language and literacy barriers	• Visual aids e.g. fats, sugars and salt pots, traffic light card.	• More visually presented information rather than reliance on written materials.
	Cultural diversity	• Target specific groups e.g. ethnicity, religion, to allow for tailoring of content and building relationships.	• More community development and linking with social housing. • Ensure service deliverers are suitably trained to deliver culturally sensitive information.
Working against the environment	Affordability; attendance and adherence	• Use health professional referrals to add value to free course. • Provide cost appropriate suggestions e.g. local deals, cheap recipes. • Linking with leisure facilities for special offers.	• Additional commitment element to course. • Considerations for policy level e.g. food vouchers.
	Access and availability	• Recommend frozen and tinned fruit and vegetables. • Suggest best options for fast food e.g. tomato rather than cream based curries. • Signposting. • Free leisure pass.	• Consideration for policy level e.g. planning. • Include strategies for replacing fast food e.g. cooking own healthier versions. • Interagency communication to identify gaps in provision.
	Life gets in the way	• Planning meals. • Damage limitation strategies e.g. knowing what not to eat at parties.	• Ensure easy to implement/ realistic goals. • Strategies to encouraging partners and families to support/ adopt changes.
